# Characterization of the T-cell Repertoire after Autologous HSCT in Patients with Ankylosing Spondylitis

**Published:** 2018

**Authors:** E. A. Komech, I. V. Zvyagin, M. V. Pogorelyy, I. Z. Mamedov, D. A. Fedorenko, Y. B. Lebedev

**Affiliations:** Shemyakin–Ovchinnikov Institute of Bioorganic Chemistry, Miklukho–Maklaya Str. 16/10, Moscow, 117997, Russia; A.A. Maximov Hematology and Cell Therapy Department, National Pirogov Medical Surgical Center, Nizhnyaya Pervomaiskaya Str. 70, Moscow, 105203, Russia

**Keywords:** autologous HSCT, TCR repertoire, NGS, ankylosing spondylitis

## Abstract

Autologous hematopoietic stem cell transplantation (HSCT), a safer type of HSCT than allogeneic HSCT, is a promising therapy for patients with severe autoimmune diseases (ADs). Despite the long history of medical practice, structural changes in the adaptive immune system as a result of autologous HSCT in patients with various types of ADs remain poorly understood. In this study, we used high-throughput sequencing to investigate the structural changes in the peripheral blood T-cell repertoire in adult patients with ankylosing spondylitis (AS) during two years after autologous HSCT. The implementation of unique molecular identifiers allowed us to substantially reduce the impact of the biases occurring during the preparation of libraries, to carry out a comparative analysis of the various properties of the T-cell repertoire between different time points, and to track the dynamics of both distinct T-cell clonotypes and T-cell subpopulations. In the first year of the reconstitution, clonal diversity of the T-cell repertoire remained lower than the initial one in both patients. During the second year after HSCT, clonal diversity continued to increase and reached a normal value in one of the patients. The increase in the diversity was associated with the emergence of a large number of low-frequency clonotypes, which were not identified before HSCT. Efficiency of clonotypes detection after HSCT was dependent on their abundance in the initial repertoire. Almost all of the 100 most abundant clonotypes observed before HSCT were detected 2 years after transplantation and remained highly abundant irrespective of their CD4+ or CD8+ phenotype. A total of up to 25% of peripheral blood T cells 2 years after HSCT were represented by clonotypes from the initial repertoire.

## INTRODUCTION

Ankylosing spondylitis (AS), also known as Bekhterev’s disease, is a chronic autoimmune disorder affecting the joints of the axial skeleton. The strong association between the risk of developing AS and the HLA-B*27 allele, as well as alleles of the other genes involved in antigen presentation to T cells, suggests that T cells are actively involved in the pathogenesis of this disease. Nonsteroidal anti-inflammatory drugs (NSAIDs) or their combinations with monoclonal antibodies, usually anti-TNFα monoclonal antibodies, are currently used to treat AS. Alas, this therapy has proved ineffective for 40% of patients [1]. Autologous hematopoietic stem cell transplantation (HSCT) has been used over the past two decades in patients with severe autoimmune diseases. This therapy has proved to be effective in patients with multiple sclerosis (MS), systemic lupus erythematosus, juvenile idiopathic arthritis, and systemic scleroderma [2-5]. The currently available data demonstrate that the clinical effect of this therapy is based on a significant reformation of the T- and B-cell repertoires as a result of deep immunosuppression, followed by the formation of a new T-cell repertoire. The pivotal role of T cells in the normal functioning and regulation of the immune system, as well as their involvement in autoimmune processes, reenforces the importance of studying the reformation of the T-cell repertoire during HSCT. Investigation of the clonal repertoire of hypervariable T-cell receptors by high-throughput parallel sequencing (Repseq) is a state-of-the-art and informative approach for monitoring the dynamics of a T-cell pool at the level of individual T-cell clones. To date, a few studies have been published on T cell repertoire reconstitution after autologous HSCT [4, 6], including two papers that analyzed the repertoire in a patient with AS after autologous HSCT [7, 8]. In this study, we performed a longer-term and more thorough investigation of the reconstitution of the T-cell repertoire in patients with AS following autologous HSCT: for the first time, we tracked the clonal dynamics of T cells during 2 years following transplantation. The cDNA barcoding technique allowed us to evaluate the clonal diversity of the repertoire in the samples more accurately than in previous studies by setting an equal analysis depth, reducing the PCR bias, and eliminating most of the incorrect sequences that emerge during PCR and sequencing.

## MATERIALS AND METHODS

**Patients**

Peripheral blood samples were collected from two patients with AS at several time points: before HSCT (point 0) and 4, 12, and 25 months post HSCT. The patients were diagnosed with AS according to the modified New York criteria [9]. All the patients provided an informed consent to participate in the study; the study was conducted in compliance with current ethical and regulatory requirements.

Patient ash-110 - a 26-year-old male, HLA-B*27+. Before HSCT, disease duration was > 3 years; the patient was treated with methotrexate (7.5 mg/week, i.m. injections) and non-steroidal anti-inflammatory drugs (NSAIDs). At the time point when HSCT was conducted, the patient had grade 2 ankylosing spondylitis and a grade 2 impaired functional status. A month after HSCT, the patient’s condition had improved and he was discharged to receive outpatient care. Acute relapse occurred one year after HSCT (point 12); after that, the patient started receiving chronic therapy with adalimumab (Humira®). Another relapse occurred two years after HSCT (point 24).

Patient ash-111 - a 28-year-old female, HLA-B*27+. Before HSCT, disease duration was > 10 years; the patient was treated with infliximab (Remicade®). At the time point when HSCT was conducted, the patient had grade 2–3 ankylosing spondylitis and a grade 2 impaired functional status. A month after HSCT, the patient’s condition had improved and she was discharged to receive outpatient care. Acute relapse occurred one year after HSCT (point 12); after that, the patient started receiving chronic therapy with etanercept (Enbrel®).

**HSCT**

Autologous HSCT was conducted according to the following protocol: immunosuppressive chemotherapy with cyclophosphamide (200 mg/kg for 4 days), followed by infusion of a cryopreserved autologous isolate of hematopoietic stem cells (2.4 × 10^6^ blood stem cells/kg body weight). The autologous stem cell transplant was mobilized using a granulocyte colony-stimulating factor (G-CSF, 10 mg/kg body weight); no CD34+ enrichment was performed. Antithymocyte globulin was transfused simultaneously with the graft to ensure *in vivo *T-cell depletion.

**Isolation of the lymphocytes and cell sorting**

Peripheral blood samples (8 ml) were collected into Vacutainer tubes with K3EDTA (BD Biosciences) 0 (before HSCT and chemotherapy), 4, 9 (ash-111) or 12 (ash-110), and 24 months post-HSCT. The mononuclear cell fraction was isolated by conventional density gradient centrifugation using Ficoll (1.077 g/cm3, PanEco, Russia). Two equal-volume samples of peripheral blood (R1 and R2) were collected from both patients to analyze the clonal repertoire reproducibility at 24 months. In the same time frame (24 months post-HSCT), individual fractions of CD4+ and CD8+ T cells were obtained simultaneously with R1 and R2 samples using the Dynabeads reagent kits for immunomagnetic separation (Invitrogen, USA).

**Preparation of TCRβ cDNA libraries and sequencing**

RNA was isolated using the TRIzol reagent (Invitrogen, USA) in compliance with the manufacturer’s protocol. The cDNA libraries were prepared using the previously published technique [10], with some modifications: after cDNA synthesis, TCR alpha and beta cDNA were pre-amplified with the primers BCuni2R TGCTTCTGATGGCTCAAACAC and M1S AAGCAGTGGTATCAACGCAGAGT (94°C, 20 s; 60°C, 15 s; 72°C, 60 s – 18 cycles). Each reaction mixture contained BCuni2R and M1S oligonucleotides (5 pmol each), 1× Tersus buffer, 0.1 mM of each dNTP, and 0.2 μl of Tersus polymerase (Evrogen, Russia); the total volume of the mixture was 15 μl. The amplification product was purified using the QIAquick PCR Purification Kit (Qiagen, USA) according to the manufacturer’s protocol. The entire purified PCR product was used for subsequent amplification.

Sequencing was carried out using an Illumina HiSeq 2000/2500 in pair-end mode with a 100 bp read length.

**Sequence data processing**

Sequencing data pre-processing included a correction of sequencing errors and counting of the number of molecular events (UMI) in the library using the MiGEC software [11]. The MiTCR software was employed to determine the V, D, and J gene segments and CDR3 sequences, to count the number of clonotypes, and to generate a list of the clonotypes identified in each sample. When reconstituting the clonal repertoire of a sample, we used the TCR cDNA sequences read at least twice according to the UMI analysis, which allowed us to eliminate most of the erroneous sequences [11, 13]. The sequencing statistics are listed in *table*. Further bioinformatic and statistical analysis of the results was performed using the R programming language and the tcR package [14, 15].

**Table:** Structures of K_V_-channels alone and in complex with charybdotoxin used in homology modeling studies

Patient	Point / T-cell population	Number of reads	Number of UMI^§,#^	Total clonotype count^#^	Clonotype count per 90,000 UMI^#^
ash-110	0/F*	10671513	301277	226383	77160
	4/F	13657155	90985	47119	46803
	12/F	1011932	125640	72925	55413
	24/R1**	7366741	964274	519425	69397
	24/R2**	7533711	1263464	625152	69244
	24/CD4+	2776546	587713	329331	71870
	24/CD8+	2964049	653168	183735	42679
ash-111	0/F	11194792	308159	222555	74115
	4/F	17725492	138299	68398	48533
	12/F	23807978	225397	134596	61615
	24/R1	7366741	957406	414203	63755
	24/R2	4597521	367849	210597	64547
	24/CD4+	3144715	435297	218230	64026
	24/CD8+	2850176	552005	163602	43519

^*^F – the fraction of peripheral blood mononuclear cells.

^**^R1, R2 – fractions of peripheral blood mononuclear cells from two parallel blood samples.

^§^UMI – unique molecular identifier.

^#^Each UMI was read at least twice.

Matching between the nucleotide sequences in the CDR3 region and the V segment of TCR in the reconstituted repertoires was employed to establish whether a clone had a CD4 or CD8 phenotype and to search for specific clonotypes in individual repertoires at different time points.

In order to calculate the clonal diversity, the estimated lower bound of frequency of the clonotypes stably detected in a sample and the degree of repertoire renewal, the analysis depth for the repertoire of the samples being compared, was aligned by random selection of 90,000 UMI from the sequence dataset for each sample.

The clonal diversity of the T-cell repertoire was evaluated using the Chao1 diversity index [16].

## RESULTS

**The dynamics of T-cell repertoire reconstitution**

When studying the changes in the T-cell repertoire by high-throughput parallel sequencing, it is very important to maximally reduce the artificial diversity effected by sequencing errors and to ensure a comparable depth of analysis for the repertoires in the samples under comparison [17]. In order to perform TCR high-throughput sequencing and reconstructions of peripheral T-lymphocyte repertoires for two AS patients before and after autologous HSCT, we applied the cDNA barcoding technique for preparing TCR cDNA libraries [18, 19]. The use of unique molecular identifiers (UMI) in the processing of the sequencing data allows one to eliminate most PCR and sequencing errors, to reduce the artificial diversity, and to quantify the frequency of each T-cell clonotype in the sample [11, 13]. Five peripheral blood samples were collected from each patient at 5 time points (one week before HSCT (point 0) and 4, 12, and 24 (a pair of parallel samples) months after HSCT). From 1 × 10^6^ to 23 × 10^6^ sequences corresponding to at least 9 × 10^4^ unique TCR β-chain cDNA molecules were obtained after sequencing of each sample; the minimal threshold was two reads per TCR cDNA molecule (*Table*). The selected threshold allowed us to eliminate most of the erroneous cDNA sequences that emerged during PCR and sequencing from further analysis [20].

We used the Chao1 index as a measure of the clonal diversity of a repertoire: this index is determined by estimating the number of low-frequency clonotypes in a sample and takes into account the richness of naïve T cell clonotypes, which underlie the diversity of the T-cell repertoire in the sample [16, 21]. Taking into account the sensitivity of this metric to the analysis depth, 90,000 TCR cDNA sequences with unique molecular identifiers were arbitrarily chosen for each sample from the dataset when we studied the dynamics of clonal diversity. Earlier evaluation of the efficiency of the technique for T-cell repertoire reconstitution used in this study demonstrated that the selected number roughly corresponds to an analysis of 90,000 T cells [13, 21].

**Fig. 1:**
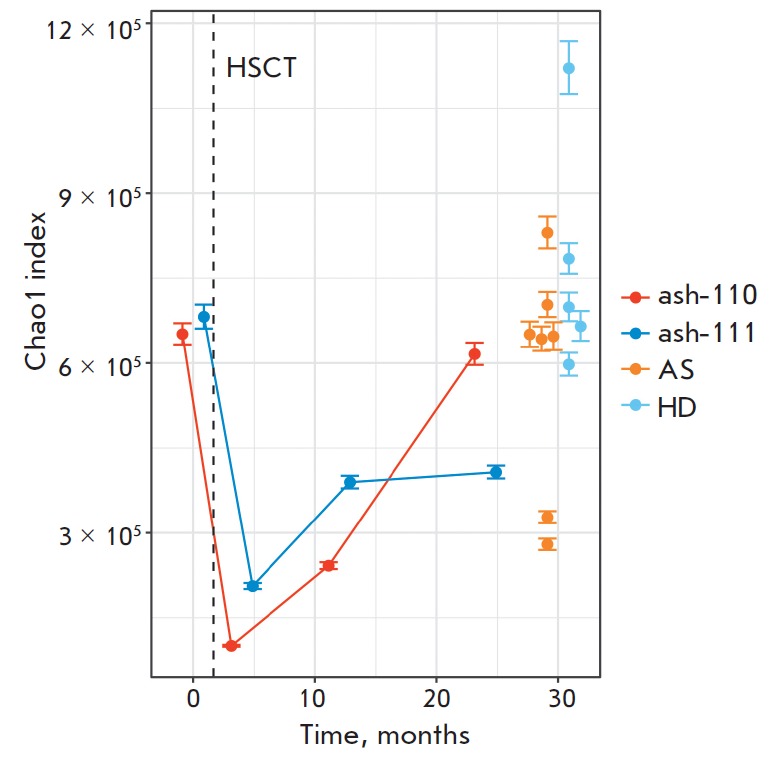
Dynamics of T-cell diversity during 2 years after HSCT. Estimation of the lower bound of clonal diversity using the Chao1 index [16]. Blue dots represent healthy donors (n=6, age 22–34 years; the data were reported by Britanova et al. 2014 [21]); orange dots – patients with AS (n=5, age 22–34; the data were reported by Komech et al. 2018 [22]). The dashed vertical line shows the time point of HSCT. The 95% confidence interval for each value is marked with an error bar.

Four months post-HSCT, the diversity (by Chao1 index) significantly decreased (*p * < 2.2 × 10-16, Mann– Whitney U test) with respect to its initial value in both patients (*Fig. 1*). The total count of the identified TCRβ clonotypes was more than twice as low as that before HSCT (77391 and 46797, 73880 and 48505 clonotypes in the samples at time points 0 and 4 months in patients ash-110 and ash-111, respectively). After the 1-year-long reconstitution period, the clonal diversity of the repertoire in both patients had not returned to its initial level. The rate of clonal diversity reconstitution was different in these two patients. Two years post-HSCT, the clonal diversity of the repertoire in patient ash- 110 had returned to its initial level and corresponded to that of healthy donors of the same age (*Fig. 1*). One year post-HSCT, the clonal diversity in patient ash-111 was only 50% of its initial value. No significant changes were observed during the second year of the reconstitution period, and a normal value was not reached. Unlike in other autoimmune diseases where the clonal diversity of the T-cell repertoire before HSCT is significantly reduced [2, 4], the initial clonal diversity of repertoires in both patients in this study was similar to that in healthy donors and/or in patients with AS of comparable age (*p *= 0.284 and *p *= 0.0, respectively, Mann–Whitney U test).

**Effect of HSCT on the clonotypes presented in the initial T-cell repertoire**

**Fig. 2:**
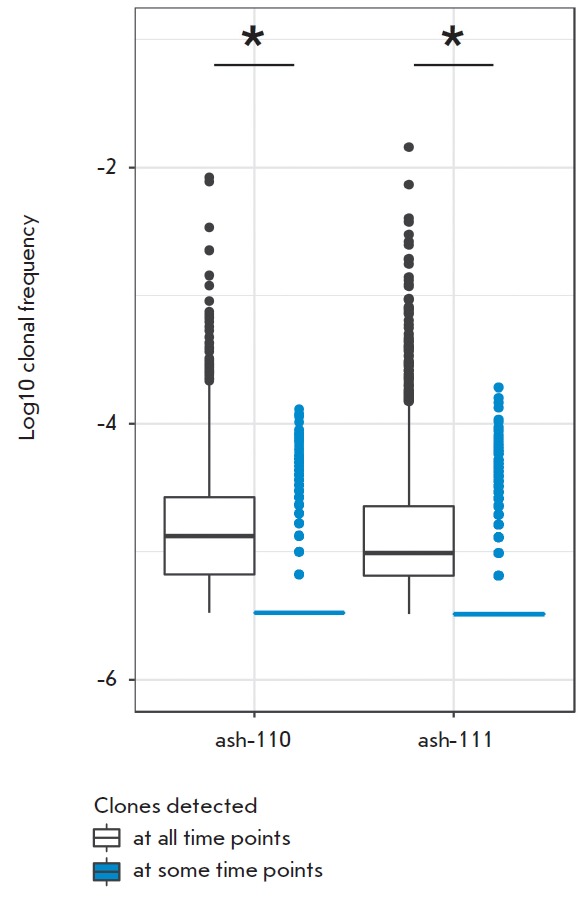
Clonal frequency distribution in the initial repertoire according to the detection of clonotypes in samples after HSCT. White box plots show the frequency distribution of clonotypes found in all the analyzed samples, including the time point before HSCT; blue box plots show the frequency distribution of clonotypes present at point 0 but not found in at least one sample after HSCT (points 4, 12 or 24). *The p-value < 2.2×10^-16^ (Mann–Whitney U-test).

When studying the dynamics of the initial repertoire during 2 years post-HSCT, we tracked the frequencies of clonotypes from the repertoire at point 0 detected in all subsequent samples (i.e., 4, 12, and 24 months post- HSCT). According to our findings, the clonotypes from the initial repertoire provisionally divided into two groups: the ones being constantly present at all points after HSCT and the ones detected at some of the subsequent time points, only. The first group consisted of 3,188 clonotypes in ash-110 repertoire and 6,126 clonotypes in ash-111 repertoire (1.4 and 3.0% of the overall diversity, respectively), corresponding to approximately 10% (ash-110) or 15% (ash-111) of peripheral blood T cells before HSCT (*Fig. 2*). The clonotypes in the second group (i.e., the ones either not detected at all or observed only at some time points post-HSCT) predominated among peripheral blood T cells before HSCT (~90%). The initial frequencies were significantly different in these groups: the first group contained most of the high-abundance clonotypes in the sampled repertoire (the median clonotype frequency in the initial repertoire was 0.001%; the interquartile range for each patient was 0.0007–0.003%), while the second group mostly contained the low-abundance clonotypes (median frequency, 0.0003%; the interquartile range was 0.0003–0.0003%). An interesting fact was that some initially low-abundance clonotypes were still detected in the first group (i.e., were present in all repertoires post-HSCT), while the high-abundance clonotypes in the initial repertoire could disappear after HSCT.

**Fig. 3:**
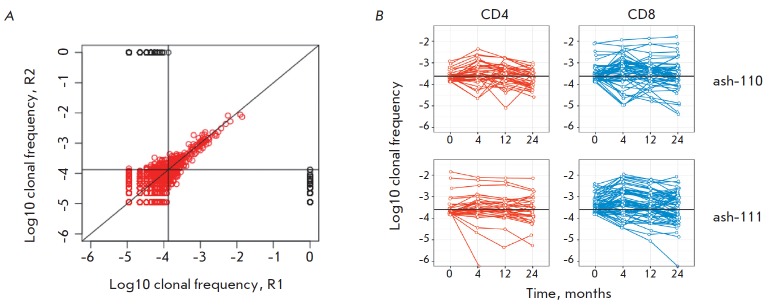
Clonal dynamics of the initial T-cell repertoire. (A) Reproducibility of clonal frequency in two parallel blood samples collected from one donor. Each dot represents a TCRβ clonotype. Black dots represent the clonotypes that do not reproduce in replica. (B) The dynamics of the top 100 clonotypes from the initial repertoire. CD4+ clonotypes are shown in red; CD8+ clonotypes, in blue. The black horizontal line represents the lower bound of clonal frequency for the top 100 clonotypes at point 24.

When studying the dynamics of individual clonotypes over a long time frame, one should take into account the fact that the probability of detecting a clonotype in a sample depends on the count of clonotype cells in the repertoire: clonotypes with a larger cell count (i.e., the so-called high-abundance clonotypes) have a higher probability of being detected in several independently collected blood samples, unlike the low-abundance ones. In order to determine the bound of abundance of clonotypes that were stably detected in the sample under analysis and were revealed in the sequencing data, we carried out a comparative analysis of the clonal repertoires of parallel blood samples collected at the same time point. In order to eliminate any influence of the sequencing dataset sizes, we randomly sampled 90,000 TCR cDNA sequences from each dataset. In both patients, only clonotypes with abundance > 0.01% (100–150 of the most high-abundance clonotypes in the repertoire) were observed in each replica at the same analysis depth (*Fig. 3A*). We used this estimation of reproducibility and tracked the 100 most high-abundance (“top 100”) clonotypes from point 0 at all time points post-HSCT in each patient to characterize the degree of renewal of the initial repertoire. We assumed that the absence or presence of these clonotypes in post-HSCT repertoires will reflect changes in the cell count for a given clonotype. In full consistency with their high abundance, all “top 100” clonotypes, except for two clonotypes in patient ash-111, proved to belong to the group of clonotypes that were constantly present in repertoires during the reconstitution period. Meanwhile, approximately 50% of the “top 100” clonotypes in each patient (45 clonotypes in ash-110 and 52 clonotypes in ash-111) remained abundant and were among the top 100 high-abundance clonotypes 2 years post-HSCT. In a similar manner, we tracked the dynamics of the 100 most high-abundance clonotypes in three healthy donors of comparable age during 2 years. In the healthy donors, who had no HSCT, 85–95% of the initial top 100 high-abundance clonotypes still remained among the “top 100” clonotypes after 2 years.

We attributed each “top 100” clonotype to subpopulations of CD8+ cytotoxic or CD4+ helper T cells by cross-analysis of the repertoires of corresponding T cell fractions. We found no significant differences in whether or not a clonotype still remained among the “top 100” clonotypes 2 years post-HSCT depending on their cytotoxic or T-helper phenotype (*Fig. 3B*).

These results allowed us to conclude that autologous HSCT did not result in complete renewal of high-abundance clonotypes in the analyzed patients: > 50% of the clonotypes remained in the repertoire and retained their high frequency. However, the HSCT-induced rearrangement of high-abundance clonotypes was more significant compared to the dynamics of the corresponding part of the repertoires in healthy donors within the same time frame. The detectability of clonotypes from the initial repertoire in post-HSCT repertoires depended mostly on clonotype abundance in the initial repertoire. Meanwhile, many clonotypes with low abundance before HSCT were still detected in the individual repertoire 2 years post-HSCT. The latter finding suggests that other factors besides initial abundance may affect the fate of T-cell clones after HSCT.

**Fig. 4:**
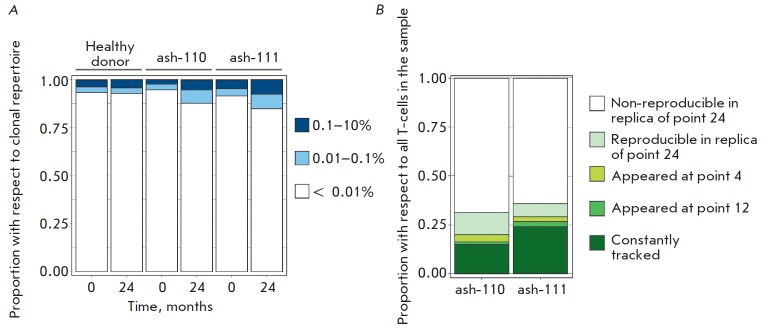
The structure of the clonal repertoire two years after HSCT. (A) Proportion of clonotypes belonging to different frequency groups with respect to the total number of clonotypes in the sample: high-abundance (0.1–10%), medium-abundance (0.01–0.1%), and low-abundance ( < 0.01%). Structures of T-cell repertoires are given for two patients with AS before and 2 years after HSCT (points 0 and 24) and for one representative healthy donor. (B) All clones at point 24 are divided into groups according to the time point when they were detected for the first time. Y-axis: the cumulative proportion of clonotypes from each group with respect to all cells in a sample 2 years after HSCT.

**Structure of the clonal repertoire 2 years post-HSCT**

Renewal of the clonal composition of the T-cell repertoire is one of the putative sequelae of HSCT determining the therapeutic potential of the procedure. In order to assess the changes that had taken place in the repertoire structure 2 years post-HSCT, we analyzed the clonal composition of the repertoire at points 0 and 24 in both patients. For the sake of comparison, we studied the repertoire structures in healthy donors of comparative age (*n*= 3) using the same analysis (*Fig. 4A*; the left panel shows the repertoire structures for one representative donor). Two years after HSCT, the structures of the clonal repertoire in patients were almost identical to the normal structure: a small fraction (up to 10%) of the repertoire was represented by high- and medium-abundance clonotypes, while the remaining fraction (approximately 90%) was represented by low-abundance clonotypes. Meanwhile, the T-cell repertoires of patients post-HSCT were more oligoclonal than before the transplantation (*Fig. 4A*).

**Fig. 5:**
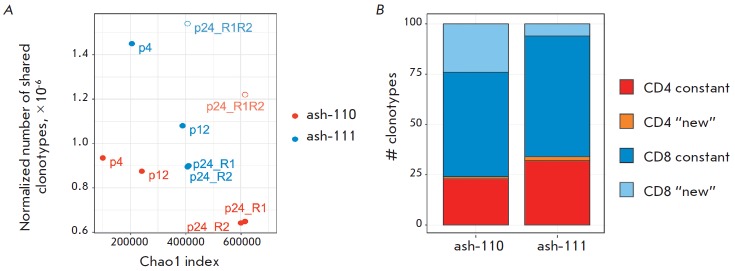
Degree of clonal repertoire renewal after HSCT. (A) Correlation between the normalized number of the clonotypes shared between point 0 and points 4 (p4), 12 (p12), 24 (in two replicas: p24_R1 and p24_R2) and the diversity of a reference sample. Points p24_R1R2 represent the comparison of replicas of point 24. (B) Composition of the 100 most abundant clonotypes in the repertoire of point 24. CD8+ cytotoxic clonotypes are shown in blue and light blue; CD4+ T-helper clonotypes are shown in red and orange.

In order to evaluate the degree of repertoire renewal, we analyzed the clonal composition 2 years post- HSCT in comparison with repertoires at all preceding time points. Fifteen and twenty-four percent (ash-110 and ash-111, respectively) of all T cells were represented by clonotypes that had been detected in the repertoire before HSCT and in all post-HSCT repertoires (*Fig. 4B*). Such clonotypes originated both from the pool of T cells that had survived in the patient’s organism after pretransplantation chemotherapy and/or from the T cells of the graft, which contains the almost complete pre-HSCT repertoire of a patient, since no specific depletion of mature T cells had been conducted. The high abundance of these clonotypes within the post- HSCT repertoires may have been caused by an intense proliferation of T cells, which would have enabled them to overcome the significant decline in lymphocyte count after pretransplantation chemotherapy. Meanwhile, in parallel with the increase in repertoire diversity, the percentage of clonotypes originated from the initial repertoire declined during 2 years post-HSCT in both patients (*Fig. 5A*). This evidence, in combination with the observation of increased clonotype sharing between replicates at point 24 (p24_R1R2 in *Fig. 5A*) in comparison with clonal sharing between point 24 and point 0, suggests that newly developed clones fill up the T-lymphocyte repertoire during the reconstitution period.

In order to analyze the degree of renewal of the repertoire of high-abundance clonotypes, we tracked the presence of the 100 most abundant clonotypes of the repertoire at point 24 in the repertoires at all earlier points. Seventy-five and ninety-two clonotypes out of 100 were detected in the pre-HSCT repertoire in patients ash-110 and ash-111, respectively (*Fig. 5B*). Among the “top 100”, 24 CD8+ and one CD4+ clonotypes in patient ash-110 and six CD8+ and two CD4+ clonotypes in patient ash-111 had not been detected before HSCT.

Hence, the fraction of medium- and high-abundance T-cell clonotypes increased and the clonal composition of low-abundance T-cell clonotypes was significantly renewed two years post-HSCT. Meanwhile, most of the high-abundance clonotypes in the 2-years-post-HSCT repertoire originated from the initial T-cell repertoire.

## DISCUSSION

Ankylosing spondylitis, also known as Bekhterev’s disease, is a chronic systemic autoimmune disorder characterized by periodic remission and relapse stages. Specific treatment for AS does not exist; disease-modifying therapy involves nonspecific nonsteroidal anti-inflammatory drugs. The conventional therapy has recently been combined with application of anti-TNFα monoclonal antibodies: infliximab (Remicaid®) and adalimumab (Humira®). However, up to 40% of patients either are resistant or stop responding to antibody-based therapy [23]. If the disease-modifying therapy proves ineffective, autologous hematopoietic stem cell transplantation can be one of the promising therapies for patients with progressive AS. Meanwhile, questions regarding the selection of an effective HSCT protocol and the overall effectiveness of this therapeutic approach are still to be answered.

Here, we continued our previous studies [7, 24] and investigated the dynamics of the clonal repertoire of peripheral T-lymphocyte during 2 years after autologous HSCT in 2 patients with AS. During the entire reconstitution period, the thymus produced new naïve T cells, thus increasing the diversity of the TCR repertoire: although it had significantly decreased short-term after HSCT, by the end of the first year the diversity of the TCR repertoire in both patients had reached 50% of its initial level and continued to accrue during the second year (*Fig. 1*). Similar dynamics of repertoire reconstitution have also been reported in adult patients with other autoimmune diseases [2, 25]. We found that the rates of clonal diversity reconstitution in the two patients differed noticeably: after 2 years, patient ash- 110 had a count of clonotypes that was nearly the same as the initial one, while the count of clonotypes in patient ash-111 was only ~50% of its initial level.

The molecular mechanisms responsible for the effectiveness of HSCT in treating autoimmune diseases remain to be elucidated. It has been demonstrated in several studies that despite chemotherapy some T cells remain within the repertoire after HSCT but have no effect on the development of stable remission of the underlying disease during 2–5 years post-HSCT [4-7]. In the patients in our study, the clones that had survived HSCT made up to 25% of the T cells within the repertoire 2 years post-HSCT. Most of the clonotypes that had survived HSCT were high-abundant clonotypes of the initial repertoire, with a mean abundance of > 0.001% (*Fig. 2*). These clonotypes could have remained in the organism after the course of chemotherapy or originated from the cells of a non-T-cell-depleted graft. Interestingly, a small fraction of initially low-abundance clonotypes was also revealed in all repertoires post-HSCT, while some high-abundance clonotypes had totally disappeared after the transplantation. In other words, clonotype survival depends not only on its abundance, but also on the functional status of *various* T cells. Kanakry et al. demonstrated that CD4+CD25+FoxP3+ regulatory T cells are resistant to medium doses (50–100 mg/kg) of cyclophosphamide *in vitro *due to an enhanced expression of aldehyde dehydrogenase, which neutralizes the cytotoxic activity of cyclophosphamide [26]. It is reasonable to assume that the low-abundant clonotypes detected after HSCT in our study were a subpopulation of regulatory T cells.

Having focused on the clonotypes stably in the samples, we followed with an assessment of the degree of renewal of the T-cell repertoire post-HSCT. In our study, approximately 50% of the clonotypes from the “top 100” ones in the initial repertoire remained in the “top 100” 2 years post-HSCT. This state was independent of whether the clonotype had a CD4+ or CD8+ phenotype. After two years, new clonotypes were detected among the “top 100” ones in both patients; these clonotypes were not detected before HSCT and mostly consisted of CD8+ T cells. In the older (45-year-old) patient with AS who had undergone HSCT according to an identical protocol and achieved long-lasting remission (> 5 years), more than one-third of the clonotypes that had been highly abundant before HSCT were still among the “top 100” clonotypes 2 years post-transplantation (data reported by Britanova et al. [7]). Hence, the patients whose T-cell repertoires were investigated in this study did not display a deep rearrangement of high-abundance T-cell clones. Reconstitution of the initial clonal structure of the repertoire could be related to the selected HSCT protocol, according to which graft preparation involved neither depletion of mature T cells nor CD34+ cell enrichment. Meanwhile, an identical HSCT protocol allowed the older patient to achieve long-lasting remission. A similar extent of renewal of high-abundance clonotypes was reported for patients with multiple sclerosis aged 27–53 years when a different protocol of autologous HSCT involving CD34+ cell enrichment was employed (about 40% of T-cell clonotypes from the “top 1000” CD4+ and CD8+ subpopulations remained within the “top 1000” corresponding fractions a year after transplantation) regardless of whether or not remission had been achieved [4]. Hence, one can only conclude that the therapeutic potential of HSCT significantly depends on other factors.

The subpopulation of regulatory T cells (T_reg_) is one of the T-cell subpopulations shown to play a crucial role in the pathogenesis of many autoimmune diseases (the data were summarized in [27, 28]). In particular, it has recently been demonstrated that renewal of this T-cell population is important for the therapeutic effectiveness of HSCT in patients with juvenile idiopathic arthritis and dermatomyositis: long-lasting remission was observed only in patients in whom HSCT had significantly increased the diversity of the clonal T_reg_ repertoire [29]. It seems reasonable to suggest that the absence of a long-term therapeutic effect by HSCT in our two patients with AS was related to insufficient reconstitution of the repertoire of the regulatory T-cell subpopulation. Further research into the dynamics of the clonal composition of various T-cell subpopulations, combined with the use of different HSCT protocols in a representative cohort of patients, will make it possible to evaluate the effectiveness of autologous HSCT as a method for treating severe forms of AS.

## CONCLUSIONS

HSCT is used increasingly often to treat patients with a severe course of autoimmune disease who fail to respond to the conventional therapy. We employed high-throughput sequencing and the cDNA barcoding technique to quantify the frequency of the clonotypes of peripheral blood T cells and, for the first time, to track the dynamics of reconstitution of the T-lymphocyte clonal repertoire during 2 years after autologous HSCT in two patients with ankylosing spondylitis. Reconstitution of the diversity of the T-cell repertoire in patients with AS lasted over two years, which is consistent with the dynamics of repertoire reconstitution in adult patients previously reported by other researchers. Two years after HSCT, up to 25% of the cells in the repertoire of the examined patients were represented by clonotypes from the pre-transplantation repertoire. We have demonstrated that almost all the high-abundance and a small fraction of the low-abundance clonotypes in the initial repertoire survived HSCT. Our findings significantly broaden the database on the functioning of the immune system upon HSCT and can be applied to optimize and elaborate new efficient protocols for autologous HSCT employed to treat severe forms of AS.

## Abbreviations

HSCT: hematopoietic stem cell transplantation
TCR: T-cell receptor
AD: autoimmune disease
AS: ankylosing spondylitis
HD: healthy donors
RepSeq: repertoire sequencing
TNFα: tumor necrosis factor alpha
